# Updated threshold dose‐distribution data for sesame

**DOI:** 10.1111/all.15364

**Published:** 2022-05-22

**Authors:** Paul J. Turner, Magdalena Gretzinger, Nandinee Patel, Helen A. Brough, R. Sharon Chinthrajah, Motohiro Ebisawa, Arnon Elizur, Jennifer J. Koplin, Rachel L. Peters, Natasha Purington, Anna Nowak‐Wegrzyn, Sarah Saf, Hugh A. Sampson, Joost Westerhout, W. Marty Blom, Joseph L. Baumert, Geert F. Houben, Benjamin C. Remington

**Affiliations:** ^1^ National Heart & Lung Institute Imperial College London London UK; ^2^ Children's Allergy Service Evelina Children's Hospital, Guy's and St. Thomas' NHS Foundation Hospital London UK; ^3^ Department of Paediatric Allergy King's College London London UK; ^4^ Sean N. Parker Center for Allergy and Asthma Research Stanford University School of Medicine Stanford CA USA; ^5^ Clinical Research Center for Allergy and Rheumatology National Hospital Organization Sagamihara National Hospital Sagamihara Japan; ^6^ Institute of Allergy, Immunology and Pediatric Pulmonology Yitzhak Shamir Medical Center Zerifin Israel; ^7^ Department of Pediatrics, Sackler Faculty of Medicine Tel Aviv University Tel Aviv Israel; ^8^ Population Health The Murdoch Children’s Research Institute Melbourne Victoria Australia; ^9^ Department of Paediatrics University of Melbourne Melbourne Victoria Australia; ^10^ Department of Medicine, Quantitative Sciences Unit Stanford University School of Medicine Stanford CA USA; ^11^ Allergy and Immunology, Department of Pediatrics New York University Langone Health New York NY USA; ^12^ Department of Pediatrics, Gastroenterology and Nutrition, Collegium Medicum University of Warmia and Mazury Olsztyn Poland; ^13^ Division of Pediatric Allergy and Immunology Icahn School of Medicine at Mount Sinai New York NY USA; ^14^ Department of Allergology‐Centre de l'Asthme et des Allergies Hôpital d'Enfants Armand Trousseau Paris France; ^15^ TNO, The Netherlands Organisation of Applied Scientific Research Utrecht The Netherlands; ^16^ Food Allergy Research and Resource Program University of Nebraska Lincoln Nebraska USA

**Abbreviations**
95% CI: 95% confidence interval
DBPCFC: Double‐blind placebo‐controlled food challenge
ED: Eliciting dose
FC: Food challenge
LOAEL: Lowest‐observed adverse effect level
NOAEL: No observed adverse effect level

To the Editor,

Sesame is classified as a “major” food allergen for which mandatory disclosure is required. Understanding reaction thresholds and how these vary within the allergic population is crucial in providing appropriate dietary advice to patients, providing guidance to the food industry, and informing dosing regimens for oral food challenges (FC). However, the largest data series used to derive a threshold dose‐distribution for sesame included blinded challenge data from just 40 individuals.^1^ Data from low‐dose, open FC can be used to supplement that from blinded FC, reducing uncertainty in estimating threshold dose‐distributions for allergenic foods which otherwise lack sufficient data.^2^ We, therefore, undertook a systematic search of the literature and performed dose‐distribution modelling of individual patient FC data (including open FC) to update estimated eliciting doses for sesame.

Eleven studies were included (Table S1), representing data from 246 positive FC. The discrete and cumulative eliciting dose predicted to provoke reactions in 5% of the sesame‐allergic population (ED_05_) were 2.4 (95% CI 1.0–7.7) and 2.5 (95% CI 0.9–9.5) mg sesame protein, respectively. Dose‐distributions are shown in Figure 1 and Table S1. These estimates are reassuringly similar to those previously reported,^1^ only with much greater precision reflecting the increased number of datapoints (Table 1). Furthermore, these estimates were robust at sensitivity analyses when excluding data from unblinded food challenges or studies with a significant proportion of “first dose reactors” (Table 1).

**FIGURE 1:**
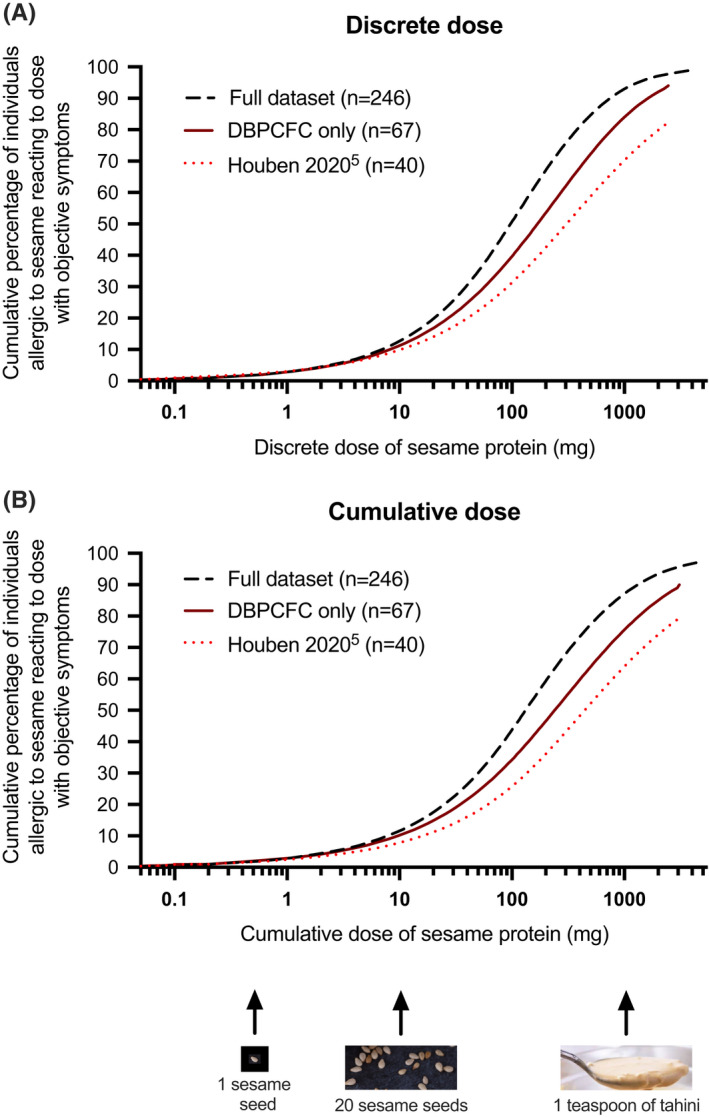
Eliciting dose curves from the model averaged population threshold dose‐distributions for sesame, based on (A) discrete and (B) cumulative dose datasets. Doses are expressed in mg sesame seed protein, and are compared to equivalent data reported by Houben et al. used to inform VITAL 3.0 reference doses^5^

**TABLE 1:** Doses of sesame protein predicted to cause a reaction in 1% (ED_01_), 5% (ED_05_) and 10% (ED_10_) of the sesame‐allergic population (together with 95% confidence intervals) calculated using both discrete and cumulative dosing schemes^a^

	ED_01_		ED_05_		ED_10_	
	Discrete	Cumulative	Discrete	Cumulative	Discrete	Cumulative
Remington et al, 2020^1^ (*n* = 40)	0.1 (0.03, 2.7)	0.2 (0.04, 4.8)	2.7 (0.4, 33.6)	4.2 (0.6, 57.7)	10.3 (1.9, 106)	16.1 (2.9, 178)
This analysis (*n* = 246)	0.2 (0.09, 1.0)	0.2 (0.08, 1.0)	2.4 (1.0, 7.7)	2.5 (0.9, 9.5)	7.0 (3.1, 19.2)	7.8 (3.1, 25.7)
This analysis (limited to DBPCFC only, *n* = 67)	0.2 (0.05, 1.8)	0.2 (0.05, 2.4)	2.6 (0.6, 17.4)	2.8 (0.6, 28.3)	8.2 (2.2, 48.7)	9.6 (2.3, 85.2)
This analysis (excluding studies with significant left‐censoring^a^, *n* = 172)	0.4 (0.15, 1.5)	0.4 (0.15, 1.6)	3.8 (1.6, 11.6)	4.2 (1.6, 14.0)	10.1 (4.5, 28.1)	12.1 (5.0, 37.0)

*Note*: Discrete dosing schemes are reported as the mg protein amount of each separate dose within a food challenge when determining the individual NOAEL and LOAEL. Cumulative dosing schemes are reported as the cumulative sum of all prior doses within a food challenge when calculating the individual NOAEL and LOAEL. Population dose‐distributions were determined using “Stacked Model Averaging” as previously described.^E2^

^a^ Left‐censoring of data occurs when participants react to the first dose of the challenge protocol, and is more likely to occur in those studies with a higher initial challenge dose. All doses are presented as mg sesame protein.

With this analysis, the dataset for sesame is now similar to that used to inform eliciting doses for other food allergens, and sufficient to inform public policy despite the potential limitations of analyses using FC data.^1^, ^2^, ^3^ The CODEX committee of the Food and Agricultural Organization of the United Nations and the World Health Organization recently commissioned an Expert Consultation which recommended the inclusion of sesame as a global “priority” allergen.^4^ The data presented here will be used to inform a reference dose which might be recommended to guide the use of precautionary allergen (“may contain”) labelling. Given that ED values remain robust at sensitivity analysis when limited to blinded FC in the ED_01_‐ED_10_ range, we recommend using ED values based on the blinded FC dataset for risk assessment and risk management purposes, to maintain consistency with approaches for other food allergens.^5^

A strength of this dataset is the inclusion of cohorts spanning four of the six global CODEX regions. These data were mostly generated from FC using ground sesame or tahini and may not be directly extrapolated to the consumption of whole sesame seeds which are commonly used in food preparation. For example, sesame seeds when baked into the surface of bread rolls are frequently not broken during mastication, and thus, swallowed whole; this prevents the release of endosperm proteins, resulting in a much lower exposure to sesame allergens. Ovadia et al. recently reported a cohort of 51 sesame‐allergic children, of whom 41 (80%) were able to tolerate 3 pretzels with sesame seeds (total exposure approximately 36 mg sesame protein) baked into the surface.^6^ This would be equivalent to an ED25 level of exposure, implying tolerance in ~25% of sesame‐allergic individuals. It is, therefore, unclear whether baked sesame seeds are tolerated due to the low level of allergen exposure, the lower bioavailability of sesame seed protein with this form of consumption, or both.

Finally, these data confirm that a semi‐log dosing regimen for FC (as recommended by PRACTALL) is appropriate for sesame. Tahini is commonly used for the higher doses used at sesame‐FC; however, the strong taste can create difficulties, particularly in younger children. Our data indicate that a top dose of 1 g protein (around 4 g of tahini paste, approximately 1 teaspoon) will cause objective symptoms in ~93% of sesame‐allergic individuals (Table S1), and thus, inform the risk of a false negative challenge in someone unable to ingest a higher dose at FC.

## FUNDING INFORMATION

RLP and JK receive research support from the National Health and Medical Research Council of Australia. NP and PJT are supported through the NIHR Biomedical Research Centre based at Imperial College Healthcare NHS Trust and Imperial College London. The views expressed are those of the author(s) and not necessarily those of the NHS, NIHR, or the Department of Health.

## CONFLICT OF INTEREST

P.J. Turner reports personal fees from Aimmune Therapeutics, Allergenis, Aquestive, and UK Food Standards Agency pe; grants from NIHR/Imperial BRC, UK Medical Research Council, UK Food Standards Agency, Jon Moulton Charity Trust, outside the submitted work. H. Brough declares speaker fees from DBV Technologies and Sanofi, and research grants from NIH (NAIAD), DBV Technologies and Aimmune Therapeutics. S. Chinthrajah reports grants from NIAID, CoFAR, Aimmune Therapeutics, DBV Technologies, Astellas, Regeneron, FARE, Stanford Maternal and Child Health Research Institute (MCHRI); other support from Alladapt Therapeutics, Novartis, Genentech, Sanofi, Allergenis, Nutricia, outside the submitted work. M. Ebisawa reports personal fees from DBV Technologies, Mylan, ARS Pharmaceuticals, outside the submitted work. J. Koplin and R. Peters receive research support from the National Health and Medical Research Council of Australia. A. Nowak‐Wegrzyn reports royalty payments from UpToDate; personal fees from the American College of Allergy, Asthma, and Immunology as Deputy Editor of the Annals of Allergy, Asthma, and Immunology; consulting fees from Nestle, Nutricia, Novartis, and Aimmune outside of the submitted work. H. Sampson reports grants from Immune Tolerance Network, NIAID/NIH, personal fees and other support from N‐Fold Therapeutics, DBV Technologies, and personal fees from Siolta Therapeutics, outside the submitted work. J Westerhout, WM Blom and GF Houben report financial support from Dutch Governmental TNO Research Cooperation Funds, Netherlands, and the Food Allergy Research and Resource Program (FARRP) of the University of Nebraska, USA. BC Remington reports grants from FARRP, grants and personal fees from DBV Technologies, and travel support from ILSI Europe, outside the submitted work. The other authors declare no competing interests.

## Supporting information


Appendix S1

## Data Availability

The data that support the findings of this study are available from the corresponding author upon reasonable request, but may be subject to non‐disclosure agreements.
