# Supplementary material for: Updated threshold dose‐distribution data for sesame
Source: Allergy. 2022 May 22;77(10):3124–62. doi: 10.1111/all.15364 (PMC9790362; doi:10.1111/all.15364)
Supplement: Supplementary file 1 — Appendix S1 [file ALL-77-3124-s001.docx]

Updated threshold dose-distribution data for sesame

Paul J Turner, Magdalena Gretzinger, Nandinee Patel, Helen Brough, R. Sharon Chinthrajah, Motohiro Ebisawa, Arnon Elizur, Jennifer J Koplin, Rachel L Peters, Natasha Purington, Anna Nowak-Wegrzyn, Sarah Saf, Hugh A. Sampson, Joost Westerhout, W. Marty Blom, Joseph L. Baumert, Geert F. Houben, Benjamin C Remington

**SUPPLEMENTARY INFORMATION**

**Supplementary Methods**

We searched Medline for articles published between January 1988 and June 2021 which described double-blind, placebo-controlled FC (DBPCFC) or open FC to sesame, using a search strategy and inclusion criteria previously described.^E1^ We also reviewed reference lists of included studies and review articles to identify other relevant studies. Authors were contacted where needed to confirm data extracted. Analyses were planned prospectively. Data relating to the No Observed Adverse Effect Level (NOAEL, highest dose with no objective symptoms) and Lowest Observed Adverse Effect Level (LOAEL, first dose with objective symptoms) were extracted in duplicate; any discrepancies identified between extracted data and published data were resolved by discussion and/or by contacting authors for clarifications. Population dose-distributions were determined using “Stacked Model Averaging” as previously described,^E2^ utilizing Bayesian stacked parametric survival methods with frailty components and interval censored failure times.

Thirteen published studies were identified for sesame FC (Table E1), representing data from 246 positive FC.^5,E3-E14^ Two studies were excluded: Li et al reported FC data from 10 adults but the starting dose, subsequent increments and interval times varied from patient to patient and are not reported;^E13^ Sokol et al reported 15 children with sesame allergy,^E14^ but only 3 underwent FC and due to unresolved data queries, were not included in this analysis.

**SUPPLEMENTARY REFERENCES**

1. Turner PJ, Patel N, Ballmer-Weber BK, Baumert JL, Blom WM, Brooke-Taylor S, et al. Peanut Can Be Used as a Reference Allergen for Hazard Characterization in Food Allergen Risk Management: A Rapid Evidence Assessment and Meta-Analysis. J Allergy Clin Immunol Pract. 2022 Jan;10(1):59-70.
2. Wheeler MW, Westerhout J, Baumert JL, Remington BC. Bayesian Stacked Parametric Survival with Frailty Components and Interval-Censored Failure Times: An Application to Food Allergy Risk. Risk Anal. 2021 Jan;41(1):56-66.
3. Brough HA, Caubet JC, Mazon A, Haddad D, Bergmann MM, Wassenberg J, et al. Defining challenge-proven coexistent nut and sesame seed allergy: A prospective multicenter European study. J Allergy Clin Immunol. 2020;145(4):1231-1239.
4. Salari F, Bemanian MH, Fallahpour M, Tavakol M, Shokri S, Baniadam L, et al. Comparison of Diagnostic Tests with Oral Food Challenge in a Clinical Trial for Adult Patients with Sesame Anaphylaxis. Iran J Allergy Asthma Immunol. 2020;19(1):27-34.
5. Saf S, Sifers TM, Baker MG, Warren CM, Knight C, Bakhl K, et al. Diagnosis of Sesame Allergy: Analysis of Current Practice and Exploration of Sesame Component Ses i 1. J Allergy Clin Immunol Pract. 2020;8(5):1681-1688.e3.
6. Yanagida N, Ejiri Y, Takeishi D, Sato S, Maruyama N, Takahashi K, et al. Ses i 1-specific IgE and sesame oral food challenge results. J Allergy Clin Immunol Pract. 2019;7(6):2084-2086.e4.
7. Appel MY, Nachshon L, Elizur A, Levy MB, Katz Y, Goldberg MR. Evaluation of the basophil activation test and skin prick testing for the diagnosis of sesame food allergy. Clin Exp Allergy 2018;48(8):1025-1034.
8. Purington N, Chinthrajah RS, Long A, Sindher S, Andorf S, O'Laughlin K, et al. Eliciting Dose and Safety Outcomes From a Large Dataset of Standardized Multiple Food Challenges. Front Immunol. 2018 21;9:2057.
9. Chan JCK, Peters RL, Koplin JJ, Dharmage SC, Gurrin LC, Wake M, et al. Food Challenge and Community-Reported Reaction Profiles in Food-Allergic Children Aged 1 and 4 Years: A Population-Based Study. J Allergy Clin Immunol Pract. 2017;5(2):398-409.e3.
10. Dano D, Remington BC, Astier C, Baumert JL, Kruizinga AG, Bihain BE, et al. Sesame allergy threshold dose distribution. Food Chem Toxicol. 2015 Sep;83:48-53.
11. Leduc V, Moneret-Vautrin DA, Tzen JT, Morisset M, Guerin L, Kanny G. Identification of oleosins as major allergens in sesame seed allergic patients. Allergy 2006;61(3):349-56.
12. Kanny G, De Hauteclocque C, Moneret-Vautrin DA. Sesame seed and sesame seed oil contain masked allergens of growing importance. Allergy. 1996 Dec;51(12):952-7.
13. Li PH, Gunawardana N, Thomas I, Ue KL, Siew L, Watts TJ, et al. Sesame allergy in adults: Investigation and outcomes of oral food challenges. Ann Allergy Asthma Immunol. 2017 Sep;119(3):285-287.
14. Sokol K, Rasooly M, Dempsey C, Lassiter S, Gu W, Lumbard K, Frischmeyer-Guerrerio PA. Prevalence and diagnosis of sesame allergy in children with IgE-mediated food allergy. Pediatr Allergy Immunol. 2020 Feb;31(2):214-218.

**Table E1**: Studies reporting food challenges to sesame

| Study | Study cohort  (positive FC only) | Food challenge protocol | Initial dose  (mg protein) | Dose interval | Number with  objective symptoms | Right censored | Left censored |
| --- | --- | --- | --- | --- | --- | --- | --- |
| Brough 2020^E3^  UK, Switzerland | 8 children  Median age 5.5y | Open FC | 30mg | 15-20min | 8 | 0 | 4 |
| Salari 2020^E4^  Iran | 16 adults (median 38y)  Median ED 133mg | Open FC  Tahini | 3mg | 15-30min | 16 | 0 | 0 |
| Saf 2020^E5^  USA | 106 children (sufficient data available for 45)  Median ED 500mg | Open FC  Tahini or seed | Variable,  ≤100mg | 15min | 45 | 0 | 17 |
| Houben 2020^5^  The Netherlands | 5 children | DBPCFC | 1.25 mg | 30min | 1 | 4 | 0 |
| Yanagida 2019^E6^  Japan | 46 children (median 6y)  Median ED 609mg | Open FC  Ground sesame in steamed cake | 38mg | 15min | 46 | 0 | 12 |
| Appel 2018^E7^  Israel | 40 children (median 7y)  12 adults (median 19y)  Median ED 180mg | Open FC | 0.3mg | 15min for ≤15mg, then 30min | 43 | 0 | 0 |
| Purington 2018^E8^  USA | 30 children & adults  Median ED 25mg | DBPCFC | 5mg | 15min | 27 | 0 | 5 |
| Chan 2017^E9^  Australia | 21 children, age 4y  Median ED 11mg | Open FC  Tahini | 69mg | 20min | 21 | 0 | 16 |
| Dano 2015^E10^  France | 9 children, 5 adults  Median ED 85mg | DBPCFC  Crushed seed | 0.85mg | 15min | 14 | 5 | 1 |
| Leduc 2006^E11^  France | 5 children, 9 adults (data available for 12)  Median ED 164mg | DBPCFC  Crushed seed | 0.85mg | 20min | 12 | 0 | 1 |
| Kanny 1996^E12^  France | 1 child, 8 adults  Median ED 1539mg | Blinded FC  Crushed seed | 1.54mg | 20min | 7 | 1 | 1 |

All doses are mg sesame seed protein. ED, eliciting dose; FC, food challenge. ^a^Right Censored: Individuals who did not objectively react to the highest dose of the progressive dosing scheme but are believed to be allergic to sesame on the basis of a strong clinical history. This individual would have an established NOAEL but not a defined LOAEL. ^b^Left Censored: Individuals reacting to the first dose of the FC, and therefore do not have an established dose where no symptoms occur, but do have a defined LOAEL.

**Table E2**: Discrete and cumulative dose dataset ED values (mg sesame protein) with 95% lower (LCI) and upper confidence interval (UCI)

|  | **DISCRETE**  **Complete dataset (n=246)** | | | **DISCRETE**  **DBPCFC only (n=67)** | | | **CUMULATIVE**  **Complete dataset (n=246)** | | | **CUMULATIVE**  **DBPCFC only (n=67)** | | |
| --- | --- | --- | --- | --- | --- | --- | --- | --- | --- | --- | --- | --- |
| **ED** | **Mean** | **LCI** | **UCI** | **Mean** | **LCI** | **UCI** | **Mean** | **LCI** | **UCI** | **Mean** | **LCI** | **UCI** |
| ED00.1 | 0.02 | 0.008 | 0.06 | 0.01 | 0.004 | 0.08 | 0.02 | 0.008 | 0.06 | 0.01 | 0.004 | 0.09 |
| ED00.2 | 0.03 | 0.02 | 0.1 | 0.02 | 0.009 | 0.2 | 0.03 | 0.02 | 0.1 | 0.02 | 0.008 | 0.2 |
| ED00.3 | 0.05 | 0.02 | 0.2 | 0.04 | 0.01 | 0.3 | 0.05 | 0.02 | 0.2 | 0.03 | 0.01 | 0.4 |
| ED00.4 | 0.07 | 0.03 | 0.3 | 0.05 | 0.02 | 0.5 | 0.07 | 0.03 | 0.3 | 0.05 | 0.02 | 0.6 |
| ED00.5 | 0.09 | 0.04 | 0.4 | 0.07 | 0.02 | 0.7 | 0.09 | 0.04 | 0.4 | 0.06 | 0.02 | 0.9 |
| ED00.6 | 0.1 | 0.05 | 0.5 | 0.09 | 0.03 | 0.9 | 0.1 | 0.05 | 0.5 | 0.08 | 0.03 | 1.1 |
| ED00.7 | 0.1 | 0.06 | 0.6 | 0.1 | 0.03 | 1.1 | 0.1 | 0.06 | 0.6 | 0.09 | 0.03 | 1.4 |
| ED00.8 | 0.2 | 0.07 | 0.7 | 0.1 | 0.04 | 1.3 | 0.1 | 0.07 | 0.7 | 0.1 | 0.04 | 1.7 |
| ED00.9 | 0.2 | 0.08 | 0.8 | 0.2 | 0.04 | 1.5 | 0.2 | 0.08 | 0.9 | 0.1 | 0.04 | 2.1 |
| ED01.0 | 0.2 | 0.09 | 1 | 0.2 | 0.05 | 1.8 | 0.2 | 0.08 | 1 | 0.2 | 0.05 | 2.4 |
| ED02.0 | 0.6 | 0.2 | 2.3 | 0.6 | 0.1 | 4.7 | 0.6 | 0.2 | 2.6 | 0.5 | 0.1 | 6.8 |
| ED03.0 | 1.1 | 0.4 | 3.9 | 1.1 | 0.3 | 8.3 | 1.1 | 0.3 | 4.6 | 1.1 | 0.2 | 12.8 |
| ED04.0 | 1.7 | 0.7 | 5.7 | 1.8 | 0.4 | 12.6 | 1.7 | 0.6 | 7 | 1.9 | 0.4 | 19.9 |
| ED05.0 | 2.4 | 1 | 7.7 | 2.6 | 0.6 | 17.4 | 2.5 | 0.9 | 9.5 | 2.8 | 0.6 | 28.3 |
| ED06.0 | 3.2 | 1.3 | 9.7 | 3.5 | 0.9 | 22.7 | 3.4 | 1.2 | 12.3 | 3.8 | 0.8 | 37.7 |
| ED07.0 | 4.1 | 1.7 | 11.9 | 4.5 | 1.2 | 28.5 | 4.4 | 1.6 | 15.4 | 5.1 | 1.1 | 48.1 |
| ED08.0 | 5 | 2.1 | 14.3 | 5.7 | 1.5 | 34.8 | 5.4 | 2 | 18.6 | 6.4 | 1.5 | 59.5 |
| ED09.0 | 5.9 | 2.6 | 16.7 | 6.9 | 1.8 | 41.5 | 6.6 | 2.5 | 22 | 8 | 1.9 | 72 |
| ED10.0 | 7 | 3.1 | 19.2 | 8.2 | 2.2 | 48.7 | 7.8 | 3.1 | 25.7 | 9.7 | 2.3 | 85.2 |
| ED11.0 | 8.1 | 3.6 | 21.9 | 9.7 | 2.6 | 56.3 | 9.2 | 3.6 | 29.5 | 11.5 | 2.8 | 99.3 |
| ED12.0 | 9.2 | 4.1 | 24.7 | 11.2 | 3.1 | 64.4 | 10.6 | 4.3 | 33.4 | 13.5 | 3.3 | 114 |
| ED13.0 | 10.4 | 4.7 | 27.5 | 12.8 | 3.6 | 72.9 | 12.1 | 4.9 | 37.6 | 15.6 | 3.9 | 130 |
| ED14.0 | 11.7 | 5.3 | 30.5 | 14.6 | 4.1 | 81.7 | 13.7 | 5.6 | 41.9 | 17.9 | 4.5 | 147 |
| ED15.0 | 13 | 6 | 33.5 | 16.4 | 4.6 | 91.1 | 15.4 | 6.4 | 46.4 | 20.3 | 5.1 | 165 |
| ED16.0 | 14.4 | 6.7 | 36.7 | 18.3 | 5.2 | 101 | 17.2 | 7.2 | 51 | 22.9 | 5.8 | 183 |
| ED17.0 | 15.8 | 7.4 | 40 | 20.4 | 5.8 | 111 | 19 | 8.1 | 55.8 | 25.6 | 6.6 | 203 |
| ED18.0 | 17.2 | 8.1 | 43.3 | 22.5 | 6.5 | 122 | 20.9 | 8.9 | 60.8 | 28.5 | 7.4 | 223 |
| ED19.0 | 18.8 | 8.9 | 46.8 | 24.8 | 7.2 | 133 | 22.9 | 9.9 | 65.9 | 31.5 | 8.2 | 244 |
| ED20.0 | 20.3 | 9.7 | 50.4 | 27.1 | 7.9 | 144 | 25 | 10.9 | 71.1 | 34.7 | 9.1 | 266 |
| ED21.0 | 22 | 10.5 | 54 | 29.6 | 8.6 | 156 | 27.1 | 11.9 | 76.6 | 38 | 10.1 | 289 |
| ED22.0 | 23.7 | 11.4 | 57.8 | 32.2 | 9.4 | 169 | 29.4 | 12.9 | 82.2 | 41.6 | 11.1 | 313 |
| ED23.0 | 25.4 | 12.2 | 61.7 | 34.9 | 10.2 | 182 | 31.7 | 14 | 88 | 45.2 | 12.1 | 337 |
| ED24.0 | 27.2 | 13.2 | 65.7 | 37.7 | 11.1 | 195 | 34.1 | 15.2 | 93.9 | 49.1 | 13.2 | 363 |
| ED25.0 | 29 | 14.1 | 69.8 | 40.6 | 12 | 209 | 36.5 | 16.4 | 100 | 53.1 | 14.3 | 389 |
| ED26.0 | 30.9 | 15.1 | 74.1 | 43.6 | 12.9 | 223 | 39.1 | 17.6 | 106 | 57.3 | 15.5 | 416 |
| ED27.0 | 32.8 | 16.1 | 78.4 | 46.8 | 13.9 | 238 | 41.7 | 18.8 | 113 | 61.7 | 16.7 | 445 |
| ED28.0 | 34.8 | 17.1 | 82.9 | 50.1 | 14.9 | 253 | 44.4 | 20.2 | 119 | 66.3 | 18 | 473 |
| ED29.0 | 36.9 | 18.1 | 87.4 | 53.6 | 15.9 | 269 | 47.2 | 21.5 | 126 | 71.1 | 19.3 | 503 |
| ED30.0 | 39 | 19.2 | 92.1 | 57.1 | 17 | 285 | 50.1 | 22.9 | 133 | 76.1 | 20.7 | 534 |
| ED31.0 | 41.1 | 20.3 | 97 | 60.9 | 18.1 | 302 | 53.1 | 24.3 | 141 | 81.3 | 22.1 | 566 |
| ED32.0 | 43.4 | 21.4 | 102 | 64.7 | 19.3 | 320 | 56.1 | 25.8 | 148 | 86.8 | 23.6 | 598 |
| ED33.0 | 45.6 | 22.6 | 107 | 68.7 | 20.4 | 338 | 59.3 | 27.3 | 156 | 92.4 | 25.2 | 632 |
| ED34.0 | 48 | 23.8 | 112 | 72.9 | 21.7 | 357 | 62.5 | 28.8 | 164 | 98.4 | 26.7 | 667 |
| ED35.0 | 50.4 | 25 | 118 | 77.3 | 22.9 | 376 | 65.8 | 30.3 | 172 | 105 | 28.4 | 703 |
| ED36.0 | 52.9 | 26.2 | 123 | 81.8 | 24.2 | 396 | 69.3 | 31.9 | 180 | 111 | 30.1 | 739 |
| ED37.0 | 55.4 | 27.5 | 129 | 86.5 | 25.6 | 417 | 72.9 | 33.6 | 189 | 118 | 31.8 | 777 |
| ED38.0 | 58.1 | 28.7 | 135 | 91.4 | 27 | 438 | 76.5 | 35.2 | 197 | 125 | 33.6 | 816 |
| ED39.0 | 60.8 | 30 | 141 | 96.5 | 28.4 | 460 | 80.3 | 36.9 | 206 | 132 | 35.5 | 856 |
| ED40.0 | 63.6 | 31.4 | 147 | 102 | 29.9 | 483 | 84.2 | 38.7 | 216 | 140 | 37.4 | 897 |
| ED41.0 | 66.5 | 32.7 | 154 | 107 | 31.5 | 506 | 88.3 | 40.4 | 225 | 148 | 39.4 | 940 |
| ED42.0 | 69.4 | 34.1 | 160 | 113 | 33 | 530 | 92.5 | 42.2 | 235 | 156 | 41.4 | 983 |
| ED43.0 | 72.5 | 35.4 | 167 | 119 | 34.7 | 555 | 96.8 | 44 | 246 | 165 | 43.5 | 1027 |
| ED44.0 | 75.7 | 36.8 | 174 | 125 | 36.4 | 580 | 101 | 45.9 | 256 | 174 | 45.6 | 1073 |
| ED45.0 | 79 | 38.3 | 182 | 132 | 38.1 | 607 | 106 | 47.7 | 267 | 184 | 47.8 | 1120 |
| ED46.0 | 82.3 | 39.7 | 189 | 139 | 39.9 | 634 | 111 | 49.6 | 278 | 194 | 50.1 | 1169 |
| ED47.0 | 85.9 | 41.2 | 197 | 146 | 41.7 | 663 | 116 | 51.5 | 289 | 205 | 52.4 | 1220 |
| ED48.0 | 89.5 | 42.6 | 205 | 153 | 43.6 | 692 | 121 | 53.4 | 301 | 216 | 54.7 | 1271 |
| ED49.0 | 93.3 | 44.1 | 213 | 161 | 45.5 | 722 | 127 | 55.4 | 314 | 228 | 57.1 | 1325 |
| ED50.0 | 97.2 | 45.6 | 221 | 169 | 47.5 | 753 | 132 | 57.3 | 327 | 240 | 59.6 | 1379 |
| ED51.0 | 101 | 47.1 | 230 | 177 | 49.6 | 786 | 138 | 59.3 | 340 | 253 | 62.1 | 1435 |
| ED52.0 | 106 | 48.7 | 239 | 186 | 51.7 | 819 | 144 | 61.4 | 354 | 267 | 64.8 | 1495 |
| ED53.0 | 110 | 50.4 | 249 | 195 | 53.9 | 854 | 151 | 63.6 | 369 | 281 | 67.5 | 1557 |
| ED54.0 | 115 | 52.1 | 258 | 205 | 56.2 | 891 | 158 | 65.9 | 384 | 296 | 70.4 | 1623 |
| ED55.0 | 120 | 53.8 | 269 | 215 | 58.6 | 930 | 165 | 68.2 | 400 | 312 | 73.5 | 1693 |
| ED56.0 | 125 | 55.7 | 280 | 225 | 61.1 | 969 | 172 | 70.7 | 417 | 329 | 76.7 | 1767 |
| ED57.0 | 130 | 57.6 | 291 | 236 | 63.7 | 1011 | 180 | 73.3 | 435 | 347 | 80 | 1844 |
| ED58.0 | 135 | 59.6 | 302 | 248 | 66.5 | 1055 | 188 | 76 | 454 | 366 | 83.6 | 1926 |
| ED59.0 | 141 | 61.7 | 314 | 260 | 69.3 | 1100 | 197 | 78.9 | 474 | 386 | 87.3 | 2012 |
| ED60.0 | 147 | 63.9 | 327 | 273 | 72.3 | 1147 | 206 | 81.9 | 495 | 407 | 91.2 | 2103 |
| ED61.0 | 154 | 66.2 | 340 | 286 | 75.4 | 1197 | 216 | 85.1 | 516 | 429 | 95.4 | 2199 |
| ED62.0 | 160 | 68.7 | 354 | 300 | 78.7 | 1250 | 226 | 88.5 | 539 | 453 | 99.8 | 2304 |
| ED63.0 | 167 | 71.3 | 369 | 315 | 82.1 | 1304 | 237 | 92.1 | 563 | 478 | 104 | 2413 |
| ED64.0 | 175 | 74 | 384 | 331 | 85.7 | 1361 | 248 | 95.9 | 589 | 505 | 109 | 2528 |
| ED65.0 | 182 | 76.9 | 399 | 347 | 89.5 | 1422 | 260 | 100 | 615 | 533 | 115 | 2651 |
| ED66.0 | 191 | 80 | 416 | 365 | 93.5 | 1485 | 273 | 104 | 644 | 564 | 120 | 2782 |
| ED67.0 | 199 | 83.2 | 433 | 383 | 97.7 | 1553 | 287 | 109 | 674 | 596 | 126 | 2922 |
| ED68.0 | 208 | 86.7 | 452 | 403 | 102 | 1624 | 302 | 114 | 705 | 631 | 132 | 3057 |
| ED69.0 | 218 | 90.4 | 471 | 423 | 107 | 1700 | 317 | 119 | 739 | 669 | 139 | >3078 |
| ED70.0 | 228 | 94.4 | 491 | 445 | 112 | 1779 | 334 | 125 | 775 | 708 | 147 | >3078 |
| ED71.0 | 239 | 98.7 | 513 | 469 | 117 | 1864 | 352 | 132 | 813 | 751 | 155 | >3078 |
| ED72.0 | 251 | 103 | 536 | 494 | 123 | 1954 | 371 | 138 | 854 | 798 | 163 | >3078 |
| ED73.0 | 263 | 108 | 560 | 520 | 129 | 2049 | 391 | 146 | 898 | 848 | 172 | >3078 |
| ED74.0 | 276 | 114 | 585 | 549 | 135 | 2150 | 414 | 154 | 945 | 902 | 182 | >3078 |
| ED75.0 | 290 | 119 | 613 | 579 | 142 | 2259 | 438 | 163 | 995 | 960 | 193 | >3078 |
| ED76.0 | 305 | 126 | 642 | 612 | 149 | 2375 | 463 | 172 | 1049 | 1024 | 205 | >3078 |
| ED77.0 | 322 | 133 | 673 | 647 | 156 | 2460 | 491 | 183 | 1108 | 1093 | 217 | >3078 |
| ED78.0 | 339 | 140 | 706 | 685 | 165 | >2462 | 522 | 195 | 1172 | 1170 | 231 | >3078 |
| ED79.0 | 358 | 148 | 742 | 727 | 174 | >2462 | 555 | 208 | 1242 | 1253 | 247 | >3078 |
| ED80.0 | 378 | 157 | 781 | 772 | 183 | >2462 | 592 | 222 | 1321 | 1345 | 264 | >3078 |
| ED81.0 | 400 | 167 | 824 | 821 | 193 | >2462 | 632 | 238 | 1406 | 1447 | 282 | >3078 |
| ED82.0 | 424 | 179 | 871 | 875 | 205 | >2462 | 676 | 256 | 1502 | 1561 | 303 | >3078 |
| ED83.0 | 451 | 191 | 923 | 934 | 217 | >2462 | 725 | 277 | 1609 | 1689 | 325 | >3078 |
| ED84.0 | 480 | 204 | 981 | 1000 | 230 | >2462 | 780 | 300 | 1731 | 1832 | 350 | >3078 |
| ED85.0 | 512 | 219 | 1046 | 1073 | 244 | >2462 | 841 | 326 | 1871 | 1995 | 378 | >3078 |
| ED86.0 | 547 | 236 | 1120 | 1155 | 260 | >2462 | 910 | 356 | 2035 | 2182 | 410 | >3078 |
| ED87.0 | 587 | 256 | 1206 | 1248 | 278 | >2462 | 990 | 390 | 2227 | 2398 | 446 | >3078 |
| ED88.0 | 633 | 277 | 1308 | 1354 | 297 | >2462 | 1081 | 429 | 2456 | 2651 | 486 | >3078 |
| ED89.0 | 684 | 301 | 1429 | 1476 | 318 | >2462 | 1187 | 474 | 2739 | 2949 | 532 | >3078 |
| ED90.0 | 744 | 328 | 1575 | 1619 | 342 | >2462 | 1312 | 527 | 3092 | >3078 | 584 | >3078 |
| ED91.0 | 814 | 359 | 1761 | 1788 | 369 | >2462 | 1463 | 589 | 3548 | >3078 | 645 | >3078 |
| ED92.0 | 898 | 395 | 2004 | 1993 | 400 | >2462 | 1648 | 661 | 4156 | >3078 | 715 | >3078 |
| ED93.0 | 1001 | 435 | 2336 | 2247 | 436 | >2462 | 1881 | 745 | 5002 | >3078 | 798 | >3078 |
| ED94.0 | 1132 | 482 | 2814 | >2462 | 478 | >2462 | 2188 | 844 | >6000 | >3078 | 895 | >3078 |
| ED95.0 | 1304 | 538 | 3544 | >2462 | 529 | >2462 | 2613 | 964 | >6000 | >3078 | 1015 | >3078 |
| ED96.0 | 1548 | 606 | >4000 | >2462 | 593 | >2462 | 3250 | 1109 | >6000 | >3078 | 1165 | >3078 |
| ED97.0 | 1932 | 693 | >4000 | >2462 | 677 | >2462 | 4342 | 1297 | >6000 | >3078 | 1364 | >3078 |
| ED98.0 | 2681 | 818 | >4000 | >2462 | 800 | >2462 | >6000 | 1566 | >6000 | >3078 | 1655 | >3078 |
| ED99.0 | >4000 | 1043 | >4000 | >2462 | 1023 | >2462 | >6000 | 2057 | >6000 | >3078 | 2187 | >3078 |
